# Identification of potential interleukin-8 inhibitors acting on the interactive site between chemokine and CXCR2 receptor: A computational approach

**DOI:** 10.1371/journal.pone.0264385

**Published:** 2022-02-24

**Authors:** Thi-Thuy-Nga Tran, Que-Huong Tran, Quoc-Thai Nguyen, Minh-Tri Le, Dieu-Thuong Thi Trinh, Khac-Minh Thai

**Affiliations:** 1 Faculty of Pharmacy, University of Medicine and Pharmacy at Ho Chi Minh City, Ho Chi Minh City, Vietnam; 2 Department of Pharmaceutical Chemistry, Da Nang University of Medical Technology and Pharmacy, Da Nang, Vietnam; 3 School of Medicine, Vietnam National University Ho Chi Minh City, Ho Chi Minh City, Vietnam; 4 Faculty of Traditional Medicine, University of Medicine and Pharmacy at Ho Chi Minh City, Vietnam; Università degli Studi di Milano, ITALY

## Abstract

Interactions between interleukin (IL)-8 and its receptors, CXCR1, and CXCR2, serve crucial roles in inflammatory conditions and various types of cancers. Inhibition of this signaling pathway has been exploited as a promising strategy in treating these diseases. However, most studies only focused on the design of allosteric antagonists-bound receptors on the intracellular side of IL-8 receptors. Recently, the first cryo-EM structures of IL-8-CXCR2-Gi complexes have been solved, revealing the unique binding and activation modes of the endogenous chemokine IL-8. Hence, we set to identify small molecule inhibitors for IL-8 using critical protein-protein interaction between IL-8 and CXCR2 at the orthosteric binding site. The pharmacophore models and molecular docking screened compounds from DrugBank and NCI databases. The oral bioavailability of the top 23 ligands from the screening was then predicted by the SwissAMDE tool. Molecular dynamics simulation and free binding energy calculation were performed for the best compounds. The result indicated that DB14770, DB12121, and DB03916 could form strong interactions and stable protein-ligand complexes with IL-8. These three candidates are potential IL-8 inhibitors that can be further evaluated by *in vitro* experiments in the next stage.

## Introduction

Interleukin (IL)-8, also known as CXCL8, was first identified as a member of the CXC chemokine family and proinflammatory protein purified from the medium of lipopolysaccharide-stimulated monocytes [1, 2]. IL-8 is first synthesized as a precursor protein of 99 amino acids and then processed by NH_2_-terminal truncation resulting in multiple isoforms. Among them, the predominant biologically active variant has 72 residues [3], present as both monomers and dimers under physiological conditions [4]. The monomer has a short loop that consists of a flexible N-terminus followed by three β-strands and a C-terminal α-helix [3]. Hydrogen bonds between the first β-strand stabilize the dimer in each molecule at a high concentration. Glutamate-leucine-arginine (ELR) sequence motif and residues near the turn at His33 are presumed to be crucial for binding to the receptor [5].

IL-8 mediates its effects via binding to two G protein-coupled receptors, CXCR1 (IL-8RA) and CXCR2 (IL-8RB), which are widely co-expressed in immune cells, such as neutrophils, monocytes, lymphocytes [5–8]. Previous studies proposed a two-site mechanism of IL-8 and its receptors interactions: between the IL-8 N-loop and the receptor N-domain residues (site 1) and between the IL-8 N-terminal and the receptor extracellular loop residues (site 2) [7, 9–16]. Moreover, while site 1 is responsible for the initial IL-8 recruitment, the interactions in site 2 are believed to play an essential role in activating CXCR2 [9, 10, 13]. Abnormal regulation of the IL8-CXCR1/2 axis was implicated in a few inflammatory-mediated diseases, such as chronic obstructive pulmonary disorders, asthma, psoriasis, or rheumatoid arthritis, where IL8 plays a vital role in the recruitment of neutrophils and other immune cells to the site of infection [17]. In addition, there is substantial evidence that IL-8 affects the growth and progression of tumor cells. Compared to normal tissues, IL-8 was overexpressed in several types of human carcinomas, including breast, colon, cervical, gastric, lung, or ovarian cancers [18]. Therefore, IL-8 appears to be a potential prognostic and predictive cancer biomarker.

Modulating IL-8 production can be an effective drug development strategy for treating inflammatory disorders and certain malignancies. Two most common approaches are direct inhibition of IL-8 receptors, CXCR1 or CXCR2 or both, and downstream regulation of IL-8 production via p38 kinase inhibition [19]. A few antibodies against IL-8 and CXCR1/2 allosteric antagonists have been studied in clinical trials. Their efficacy, however, is yet to be proved [17]. Since there had been no structure IL-8 and its receptors complexes, there are limited studies on IL-8 inhibitors based on active site interaction. In 2020, the first cryo-electron microscopy (cryo-EM) structures of both dimeric and monomeric IL-8 bound CXCR2-Gi complexes were solved, shedding more light on the interaction between IL-8 and its receptor at site two [13].

This study aimed to identify small molecule IL-8 inhibitors utilizing *in silico* approach: *(i)* Structure-based pharmacophore models were generated from specific residues of IL-8/CXCR2 complex. These models were used to virtually screen two databases: the DrugBank and the National Cancer Institute (NCI) database. *(ii)* Subsequently, the molecule docking was performed to determine the small molecule’s conformations and its position within the active site and assess the binding potential. *(iii)* Top compounds were validated by ADME analysis, molecular dynamics simulations, and free binding energy.

## Materials and methods

### Protein structures

Both the cryo-EM structure of the monomeric IL-8 bound CXCR2-Gi complex (PDB ID 6LFO) and the crystallographic structure of IL-8 (PDB ID 4XDX) are available for downloading from the Protein Data Bank [20].

### Database of small molecules for virtual screening

The library of 295,348 compounds from the DrugBank [21] and the National Cancer Institute chemical database [22] was used for virtual screening to discover potential IL-8/CXCR2 interaction inhibitors.

The molecules in the two databases were first filtered through the “Lipinski’s rule of five” [23] in the Molecular Operating Environment (MOE) 2015.10 software [24]. This screening resulted in 259,692 compounds, which were then conformationally searched using the conformational Import tool in MOE 2015.10 software. The setting parameters were as follows: stochastic search iteration limit was 1000, energy minimization iteration limit was 1000, energy minimization gradient test was 0.0001, and other parameters were at default [25]. Finally, the output conformers were saved as MOE database (*.mdb).

### Pharmacophore model generation

Structured-based pharmacophore (SBP) designing develops pharmacophores by using the structure of the target protein (PDB file) at the specified binding site [26–28]. From the recently solved 3D structure of the human IL-8-CXCR2-Gi complex, there are two binding sites between IL-8 and CXCR2, of which site 2 is believed to play a vital role in the activation of the CXCR2 receptor [13]. The hot-spot residues of IL-8 and CXCR2 contributing to binding site 2 were also proven by mutation experiments [13, 29]. In detail, Glu4, Leu5, and Arg6 were known to be essential for receptor binding. Individual mutations of Glu4, Leu5, or Arg6 to Ala had approximately 100, 100, and 1000-fold reduced receptor binding affinity, respectively [29]. Similarly, the corresponding critical residues of CXCR2 (Arg208, Arg212, Arg278, Tyr197, and Thr285) were also found to be necessary for IL-8 interaction and activity (Table 1) [13].

**Table 1:** Interactions between the residues of IL-8 and the corresponding residues of CXCR2 receptor in the active site 2.

IL-8 residues	Type of interaction	CXCR2 residues
Glu4^a^	(negative) electrostatic interactions (positive)	Arg208 ^a^, Arg212 ^a^, Arg278 ^a^
	(acceptor) hydrogen bond (donor)	Tyr197 ^a^
Leu5 ^a^	hydrophobic interactions	Val187, Val 192
	(donor) hydrogen bond (acceptor)	Tyr197 ^a^
Arg6 ^a^	(donor) hydrogen bond (acceptor)	Thr285
Gly31	(donor) hydrogen bond (acceptor)	Thr204
Pro32	(donor) hydrogen bond (acceptor)	Thr204

^a^ Site-directed mutagenesis of residues of IL-8 and CXCR2 have identified them as being necessary for binding.

Two strategies to construct the SBP modeling have been applied in this study. From the IL-8/CXCR2 complex, the first pharmacophore model was built using the potential protein-protein interactions [26, 30–34]. The key pharmacophoric features that mimic the components of the CXCR2 receptor are easily determined from the characterization and position of specific residues, including Arg208, Arg212, Arg278, Tyr197, and Thr285. The second pharmacophore model was generated based only on IL-8 active site information [26, 33]. The residues on the surface of the binding site 2 of IL-8 were identified as Glu4, Leu5, Arg6, Gly31, and Pro32. [13]. Hence, the 3D-pharmacophore model is constructed based on the chemical features of these residues and spatially favorable locations of their functional groups [33].

### Molecular docking

#### Protein and ligand preparation

The IL-8 structure (PDB ID 4XDX) used for docking calculations was initially prepared by the MOE software. In particular, setting steps comprised of protonating and charging amino acids (protonate), minimizing energy (tether and minimize), and converting to *.pdb format. The ligand for docking was performed energy minimization by MOE. The results were converted and saved as a *.sdf file [24].

#### Docking experiment

Docking calculations were carried out with the program LeadIT (version 2.1.8), developed by BioSolveIT, including the docking tool FlexX [35]. The protein was loaded into LeadIT, after which amino acids Glu4, Leu5, Arg6, Gly31, and Pro32 within a radius of 6.5 Å were selected in the binding pocket. Docking runs were operated by using the following parameters: the maximum number of solutions per iteration was 1000, that per fragmentation was 200, the number of poses to keep was the top 10, and default docking options were used. Successfully docked ligand conformations were evaluated by docking scores and the binding with targets, especially with crucial residues.

### ADME analysis

The accessible SwissADME web server [36] was used to predict the ADME values of best ligands after molecular docking by loading the ligand files directly in *.smiles format onto the submission page (http://www.swissadme.ch). ADME parameters are in the optimal range for each property. In detail, the molecular weight is between 150 and 500 g/mol. A compound with no more than ten rotatable bonds and twelve H-bond will have a high probability of good oral bioavailability. The topological polar surface area (TPSA) value should be between 20 and 130 Å. For lipophilicity, we used the descriptor Consensus *LogP*_(*o*/*w*)_, the arithmetic mean of the *logP*_(*o*/*w*)_ values predicted by the five proposed methods. A drug’s solubility is better when LogP is less than +5. SwissADME provides three methods to predict water-solubility and qualitative solubility classes. The ESOL model is a fast and robust method for estimating the solubility without recourse to physical measurements, where logS should be from −6.5 to 0.5 mol/dm^3^. The BOILED-Egg plot indicates biological barrier crossing such as absorption and brain access [36–38].

### Molecular dynamics simulations

MD simulations were performed using the Gromacs 2020.2 software to evaluate the stability and conformational flexibility (global and local) of the protein and ligand [39]. The simulations were carried out for the apoprotein state and the ligand-protein complexes. Protein topology was prepared by the pdb2gmx module of GROMACS using the all-atom CHARMM27 force field. Ligand topologies were constructed by the Swissparam web server [40] after adding hydrogen atoms to the initial structure by the Avogadro software [41]. The generated GROMACS compatible files for the proteins and the ligands were then merged, solvated, minimized, and equilibrated. Production runs of 50 ns were performed at 300K for the NVT (isothermal-isochoric) ensemble and 1.0 bar pressure for the subsequent NPT (isothermal-isobaric) ensemble. Trajectory snapshots were stored at every 10 ps during the simulation and later analyzed to evaluate the deviation of structures from the initial configuration through the root mean square deviation (RMSD), the mobility of amino acids via the root mean square fluctuations (RMSF), and the occupancy interactions between ligands and crucial residues (%Occupancy) by the equation as follows:

$$\%Occupancy_{i}=\left[\frac{1}{N}\overset{N}{\underset{x=1}{\sum }}\left(C_{i}\left(t_{x}\right)\right)]x100\%\right]x100\%$$ (1)

where N is the number of the trajectory’s frames sampled in the MD simulations, C_i_ is the total number of contacts of residue i with the ligand in frame x. Occupancy proportion values of some protein residues can be >100% as they form multiple contacts of the same subtype with the ligand.

### MM/PBSA binding energy calculation

In drug design, the binding free energy often characterizes the binding strength between a protein and a drug molecule. Binding free energy was determined using the molecular mechanics Poisson–Boltzmann surface area (MM/PBSA) method with the following equations:

$$\Delta G_{bind}=G_{complex}-\left(G_{protein}+G_{ligand}\right)$$ (2)

$$\Delta G_{bind}=E_{MM}+G_{sol}-\text{T}\Delta S$$ (3)

$$E_{MM}=E_{bond}+E_{nonbond}=E_{int}+E_{vdw}+E_{ele}$$ (4)

$$G_{sol}=G_{pol}+G_{nonpol}$$ (5)

Total free energies of the protein-complex, protein, and ligand are indicated by *G*_*complex*_, *G*_*protein*_ and *G*_*ligand*_, respectively. The Δ*G*_*bind*_ was decomposed to its individual contributions (Eqs 3 to 5). The vacuum potential energy (*E*_*MM*_) is a sum of bonded (*E*_*int*_) and nonbonded terms, including both *E*_*ele*_ (electrostatic) and *E*_*vdw*_ (van der Waals) energies. Similarly, the solvation free energy (*G*_*sol*_) was decomposed into polar (*G*_*pol*_), nonpolar (*G*_*nonpol*_) solvation energy components, and the conformational entropy (-TΔS). In the MM/PBSA approach, the entropy contribution can be omitted if relative binding free energies of different ligands for the same protein shall be computed [42–45]. The bonded terms can get canceled due to the single trajectory approach used. Hence, the binding free energy was calculated as the sum of van der Waals energy, electrostatic energy, polar solvation energy, and solvent-accessible surface area energy (SASA) [46].

## Results and discussion

### Pharmacophore models

**Fig 1:**
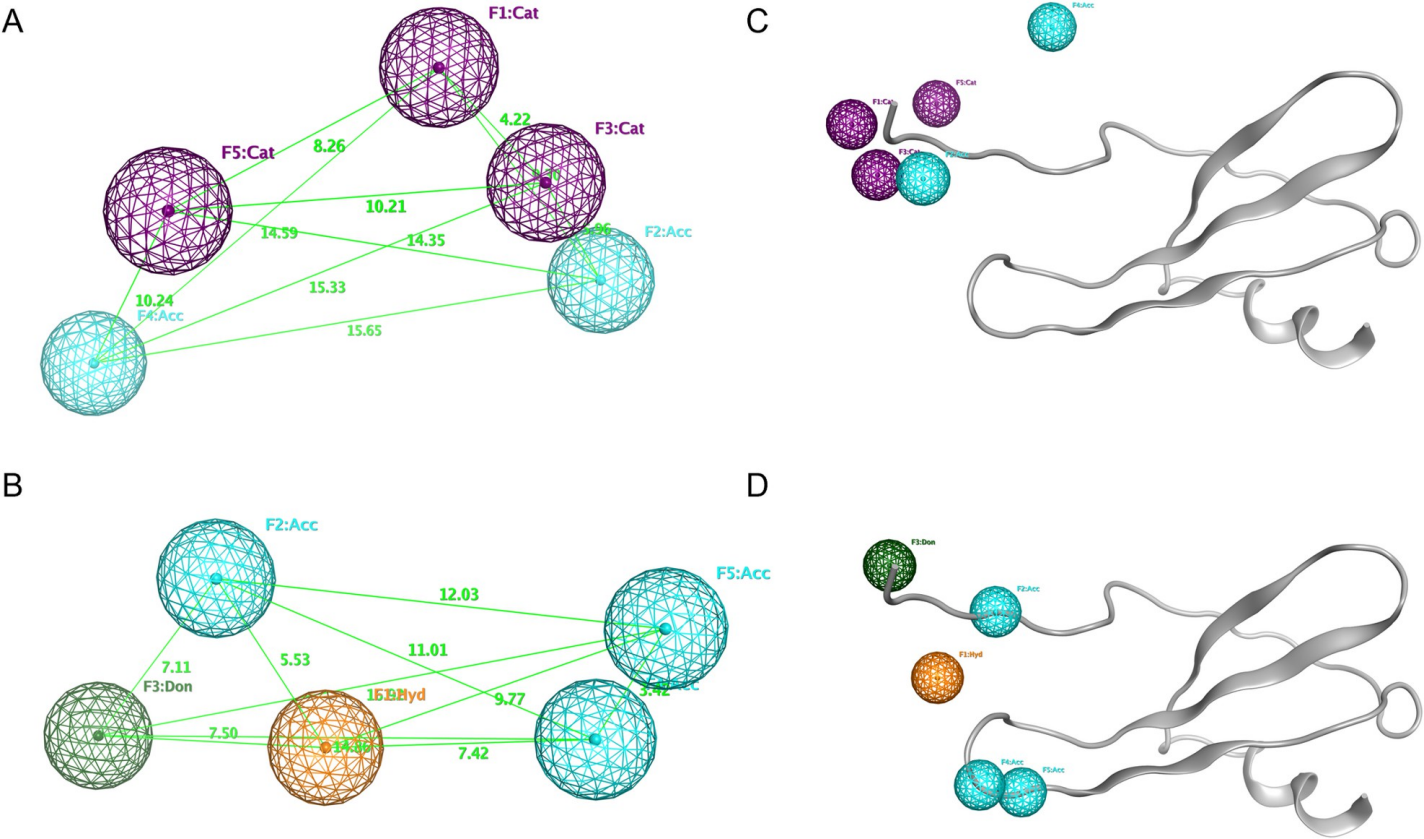
The generated pharmacophore models and their alignment to the IL-8 backbone. (A-B) The pharmacophore models were constructed by two SBP strategies. (C-D) Two of them were mapped on the IL-8 structure (shown as grey ribbons). Pharmacophore features were color-coded spheres: purple (cat), cyan (acc), dark green (don), orange (hyd). The light green lines indicated the distance (Å) between pharmacophore points.

Based on the structure-based approach, two pharmacophore models were constructed by the Pharmacophore Query Editor tool that can be directly mapped onto IL-8’s structure. In the first pharmacophore model (Ph-1), the features were represented as five points from the residues of the CXCR2 receptor that interacted with IL-8 at the binding site. In detail, Arg 208, Arg212, Arg278 of the receptor interact with Glu4 of IL-8, Tyr197, and Thr285 of CXCR2 form hydrogen bond with Leu5 and Arg6 in the “ELR” motif of IL8, respectively [13]. The developed pharmacophore contained F1: Cat (cationic), F2: Cat, F3: Cat, F4: Acc (hydrogen acceptor), and F5: Acc. Nevertheless, it is known that drug candidates as small molecules are unlikely to satisfy the pharmacophore model containing excessive charged points [33]. Therefore, three cationic points (F1, F2, and F3) were constrained to “at least one” property, and two points (F4 and F5) were essential features (Fig 1A and 1C).

The second pharmacophore model (Ph-2) comprised five features that must complement to functional groups of Glu 4, Leu5, Arg6, Gly31, and Pro32 of IL-8. For instance, the −COOH group in IL-8’s Glu4 considered by default as hydrogen acceptor would be converted to hydrogen donor and labeled out as a donor pharmacophore feature. Thereby, the pharmacophore model was manually generated with five points as follows: one hydrophobic point (F1: Hyd), three hydrogen bond acceptor points (F2, F4, F5: Acc), and one hydrogen bond donor feature (F3: Don). Because the ELR motif in the N-terminal peptide is a crucial residue, F1, F2, F3 were essential points, and either F4 or F5 was constrained at least one point in this model (Fig 1B and 1D).

### Molecular docking model

**Fig 2:**
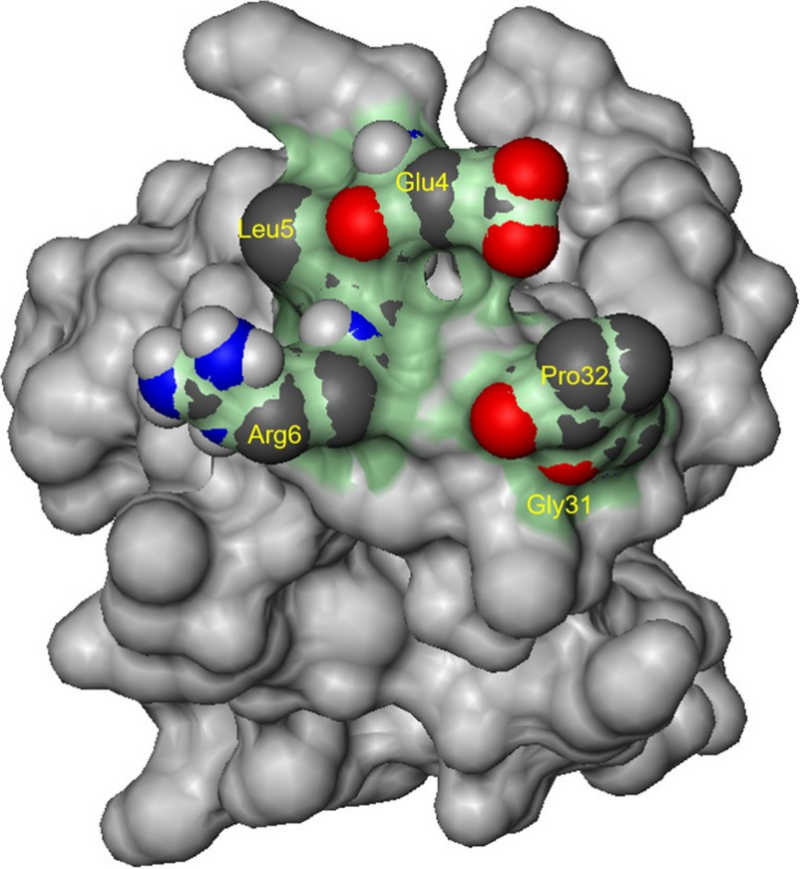
The molecular docking model. Glu4, Leu5, Agr6, Gly31, and Pro32 of IL-8 were shown as spheres, the molecular docking model as the green region, and the remaining residues of IL-8 in grey.

It was known that Glu4, Leu5, Arg6 residues (“ELR” motif) on the surface of IL-8 were important for receptor binding. In addition, the “GP” motif (Gly31, Pro32) of the 30s-loop and the conserved N-terminal “ELR” motif of IL-8 formed significant intermolecular interactions with CXCR2 [13]. Hence, interactions within this area were suitable to construct a molecular docking model (Fig 2). Since these residues were on the surface, small ligand binding grooves in this model were not too deep.

### *In silico* screening

**Fig 3:**
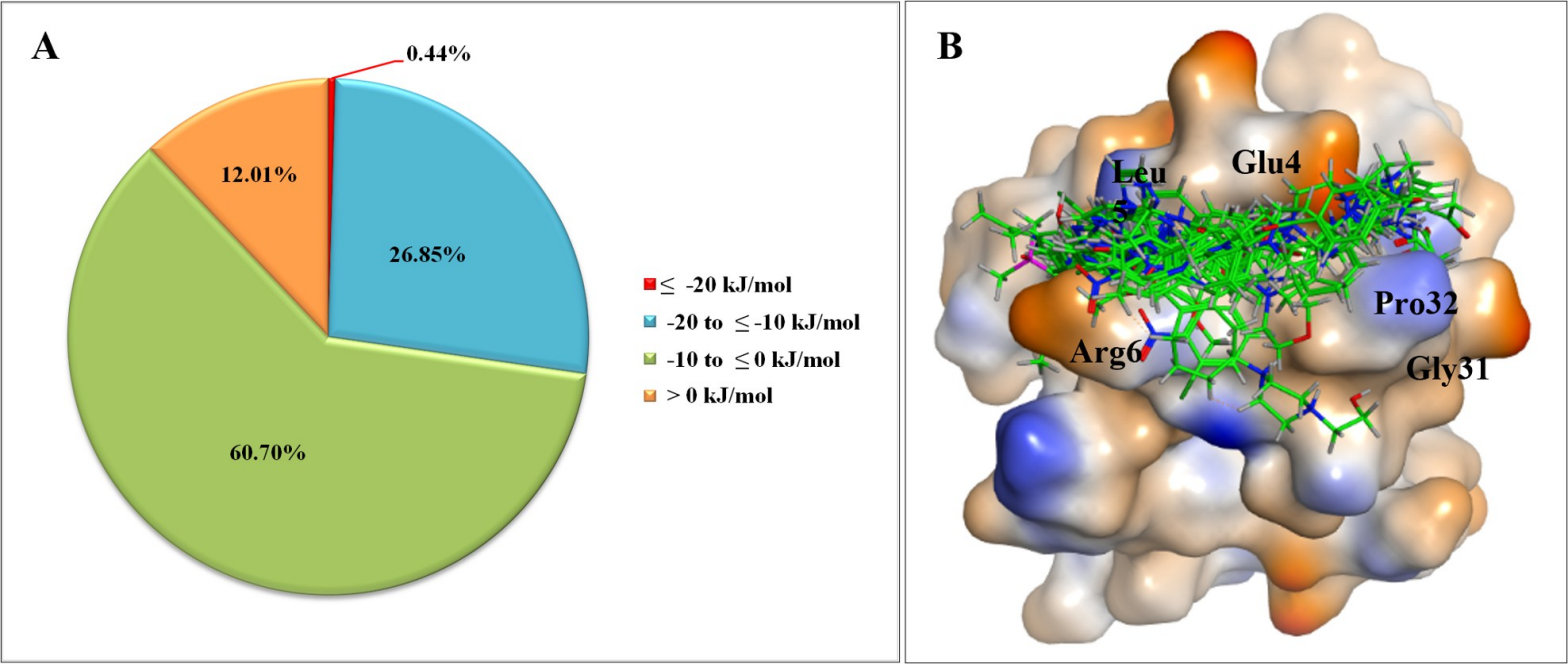
Results of molecular docking. (A) Percentage of docked ligands into the binding pocket of IL-8 according to the score range. (B) The top 23 hit compounds (carbon atoms in green) in the binding pocket.

The constructed pharmacophore models were used to screen 259,692 compounds previously prepared for conformational sampling from the DrugBank and NCI databases. This screen resulted in 5,967 compounds that fulfilled the total two pharmacophore models’ constraints. They were ranked based on their RMSD values. After being further refined with RMSD and energy minimization, the hit substances were used for docking into the binding pocket of IL-8 (Fig 2). As a result, 5,718 compounds successfully docked to IL-8; 0.44%, 26.85%, and 60.70% of which had docking scores <−20 kJ/mol, from −20 to −10 kJ/mol and from −10 to 0 kJ/mol, respectively. The remaining compounds had positive docking scores (12.01%) (Fig 3A). Top 23 ligands neatly bound to the specified binding pocket on IL-8 with the presence of the five residues were shown in Fig 3B, and their docking scores listed in Table 2.

**Table 2:** Studied ligands with docking scores smaller than −20kJ/mol.

Rank	Ligand ID	Docking Score (kJ/mol)	IL-8 residues interaction	Satisfying pharmacophore models
1	NCI640971	−23.8640003	Glu4, Leu5, Arg6	Ph-2
2	NCI640965	−22.7520008	Glu4, Leu5, Arg6, Ile28	Ph-2
3	DB13060	−22.2259998	Leu5, Arg6, Gly31, Pro32, His33	Ph-1 and Ph-2
4	DB12121	−21.7240009	Glu4, Leu5, Arg6, Gln8, Pro32	Ph-1
5	NCI144941	−21.9069996	Glu4, Leu5, Gly31, Pro32, Gln8, Cys7	Ph-1
6	NCI65378	−21.6900005	Glu4, Leu5, Arg6	Ph-1
7	NCI641429	−21.6539993	Glu4, Leu5, Arg6, Gly31, Pro32	Ph-2
8	NCI53309	−21.5330009	Glu4, Leu5, Arg6, Gln8, Cys7	Ph-1
9	NCI630293	−21.4549999	Glu4, Leu5, Arg6, Pro32, Cys7	Ph-2
10	NCI641442	−21.3889999	Glu4, Leu5, Arg6, Pro32, His33	Ph-2
11	NCI673841	−21.3630009	Leu5, Arg6, Pro32	Ph-1
12	DB14770	−20.9659996	Glu4, Leu5, Arg6	Ph-1
13	NCI658915	−20.9230003	Glu4, Leu5, Arg6, His33	Ph-2
14	DB03916	−20.8980007	Glu4, Leu5, Arg6, Gln8	Ph-1
15	NCI63667	−20.6219997	Glu4, Leu5, Arg6, Pro32, Cys7	Ph-1
16	NCI641433	−20.5839996	Glu4, Leu5, Arg6, His33	Ph-2
17	NCI89682	−20.4300003	Glu4, Leu5, Arg6, Pro32, His33	Ph-1
18	NCI71041	−20.3929996	Glu4, Leu5, His33, Ile28, Gln8, Arg26	Ph-1
19	NCI106128	−20.3780003	Glu4, Leu5, Arg6, His33, Lys3	Ph-2
20	NCI270335	−20.1660004	Glu4, Leu5, Arg6, Ile28, His33, Glu38	Ph-1
21	DB12267	−20.1590004	Glu4, Leu5, Arg6	Ph-1 and Ph-2
22	NCI106112	−20.0939999	Leu5, Arg6	Ph-2
23	NCI640966	−20.0330009	Glu4, Leu5, Arg6	Ph-2

Out of 23 compounds, twenty interacted with three key residues and other amino acids of IL-8. The number of substances that satisfied either the Ph-1 or the Ph-2 model was roughly equal, among which DB13060 and DB12276 satisfied both models. The detailed interaction models of the top 23 compounds with the residues in the binding pocket of IL-8 were shown in S1 Fig.

### ADME analysis

The ADME parameters of the SwissADME web tool can be used to estimate various characteristics of small molecules, such as physiochemistry, pharmacokinetics, drug-likeness, medicinal chemistry friendliness, and related properties [36]. Compounds that do not meet the criteria of this “drug-like” analysis will be omitted for MD simulations.

Physicochemical and solubility descriptors for the top ligands were shown in the S1 Table. Most top docked compounds fell in the optimum range for drug-likeness except for DB13060 and DB2276 (MW > 500). Although the majority of substrates had no more than ten rotatable bonds, several of the compounds, namely NCI640971, NCI640965, NCI641429, NCI630293, and NCI641442, could not have good oral availability because they had 11 rotatable bonds. The numbers of H-bond acceptors and donors of the ligands were in the range of 4–8 and 2–6, respectively. The ESOL LogS values of four ligands, NCI89682, NCI71041, NCI106128, and NCI270335, were out of standard range and highly soluble.

**Fig 4:**
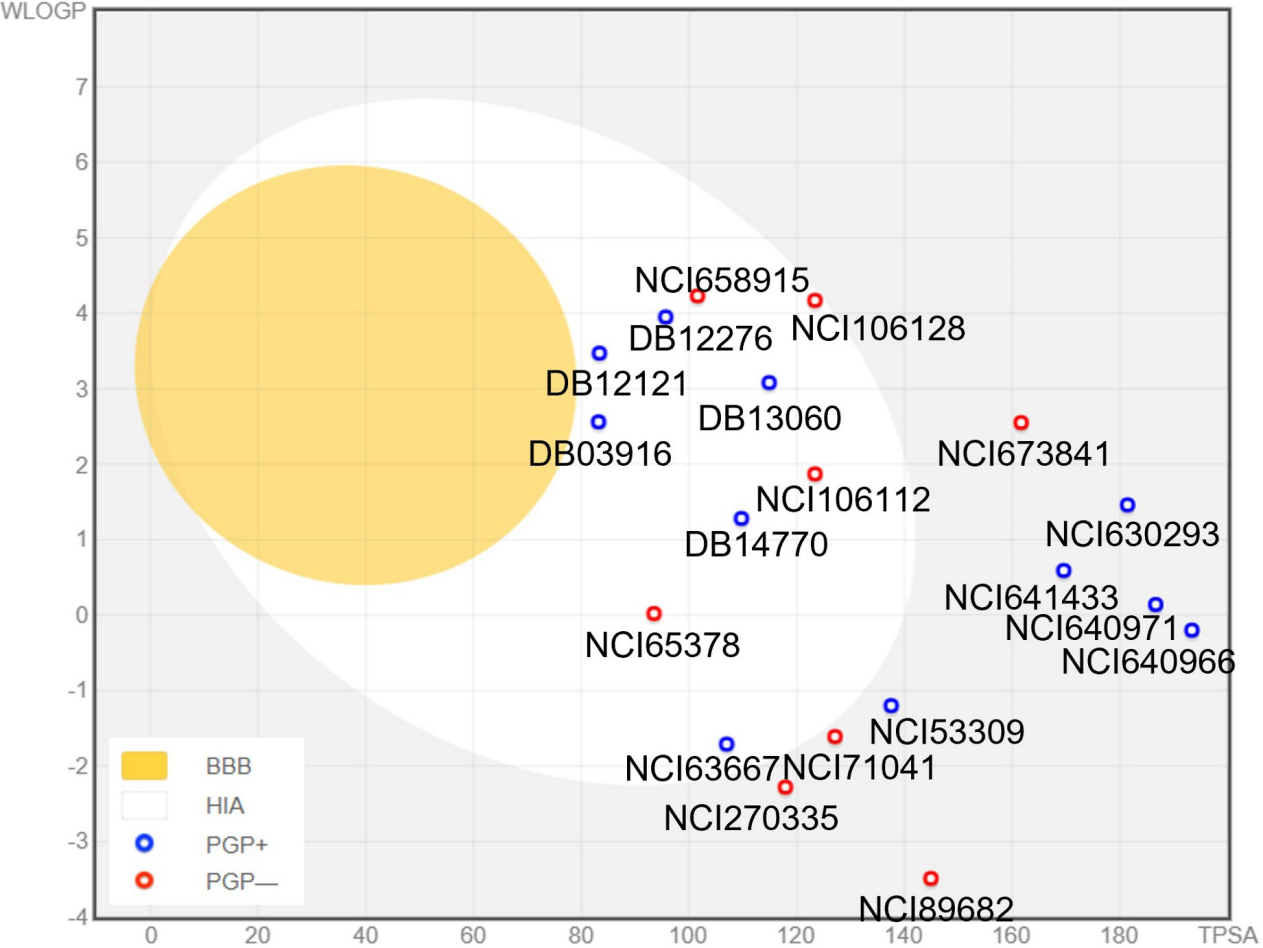
TPSA and WLOGP of top ligands plotted on the BOILED-Egg.

The BOILED-Egg plot (Fig 4) to predict the molecules’ oral bioavailability relied on WLOGP and TPSA values (for lipophilicity and polarity). There were four substances with TPSA values that were greater than 200 Å and were not shown on this plot. The grey space was associated with minimal human intestinal absorption (HIA) and restricted blood-brain barrier (BBB) permeation. In contrast, the white egg region was associated with passive HIA without brain penetration. Furthermore, the chemical was located within the yolk area, allowing it to cross through the BBB. Even though NCI106128 was on the line between white and grey, it nevertheless had a low bioavailability profile. Thus, nine compounds only in the white egg region would be suitable for drug development. In the graph, the blue dots were for P-GP substrates (PGP+) and red dots for P-GP non-substrate (PGP−). More than half of the candidates were P-GP substrates, suggesting that they can be actively effluxed from the brain or the gastrointestinal lumen. This could lead to decrease intestinal absorption and molecule’s bioavailability, presumably protecting the central nervous system [36].

Drug metabolism via the cytochrome P450 (CYP) system, especially five major isoforms (CYP1A2, CYP2C19, CYP2C9, CYP2D6, CYP3A4) play a significant role in drug interactions that can result in toxicities, reduced pharmacological effect, and adverse drug reaction. We found that more than half of top virtual screening hits could potentially cause severe drug-drug interactions by inhibiting three to five isozymes. Meanwhile, a few ligands did not affect the CYP450 system (S2 Table).

### Molecular dynamics simulations

The best seven molecules were selected from the docking to validate protein-ligand binding with 50 ns MD simulations, including DB12121, DB14770, DB03916, NCI65378 NCI658915, NCI63667, and NCI106112. The simulation trajectories were analyzed to predict spatial fluctuations of the apoprotein and the protein-ligand complex.

#### Stability of the structures

The RMSD of carbon backbone atoms was calculated for the apo and the complex states, as presented in S2 Fig. According to Roccatano [47], depending on the size of the protein, *C*_*α*_ RMSD values within 2 Å are usually considered acceptable, whereas in Reva’s study [48] this value was less than 3 Å for two 100-residue chains. The RMSD range was found to be about 1–3.3 Å for the apostate, slightly high in the beginning but becoming less flexible after 10 ns till the end of the simulation. When complexed with NCI658915, both the protein and the ligand became very unstable during the simulation with a backbone deviation range of about 4 Å. On the other hand, DB14770 and NCI65378 in the complex had the lowest RMSD with a fluctuation value < 1 Å, while DB03916 and DB12121 complexes had constant RMSD values over the simulation period. NCI106112 remained its stability for a long time and only deviated largely from its initial position at the last ten ns with an RMSD value in a range of 2 Å. From this analysis, it could be concluded that most ligands binding to IL-8 played a role in the structural stability of the protein.

The root mean square fluctuation (RMSF) is a measure of the mobility of the alpha carbons of the protein chain. Residues bearing RMSF values below 2 Å are considered to reach a steady state during the simulation [49]. Most RMSF results of the apoprotein and seven complexes were quite similar in this simulation, ranging from 0.6 to 1.6 Å (S3 Fig). Inspection of the EFR motif, which is crucial for protein interaction, revealed that RMSF of Glu4 of both apoprotein and complexes were above 2 Å, whereas that of Leu5 and Arg6 did not exceed 2 Å. A few atoms at the N and C termini were more mobile than the remaining molecule because these flexible regions may be involved in intermolecular interactions. This result suggested that protein-ligand complexes were relatively stable at the binding site.

**Fig 5:**
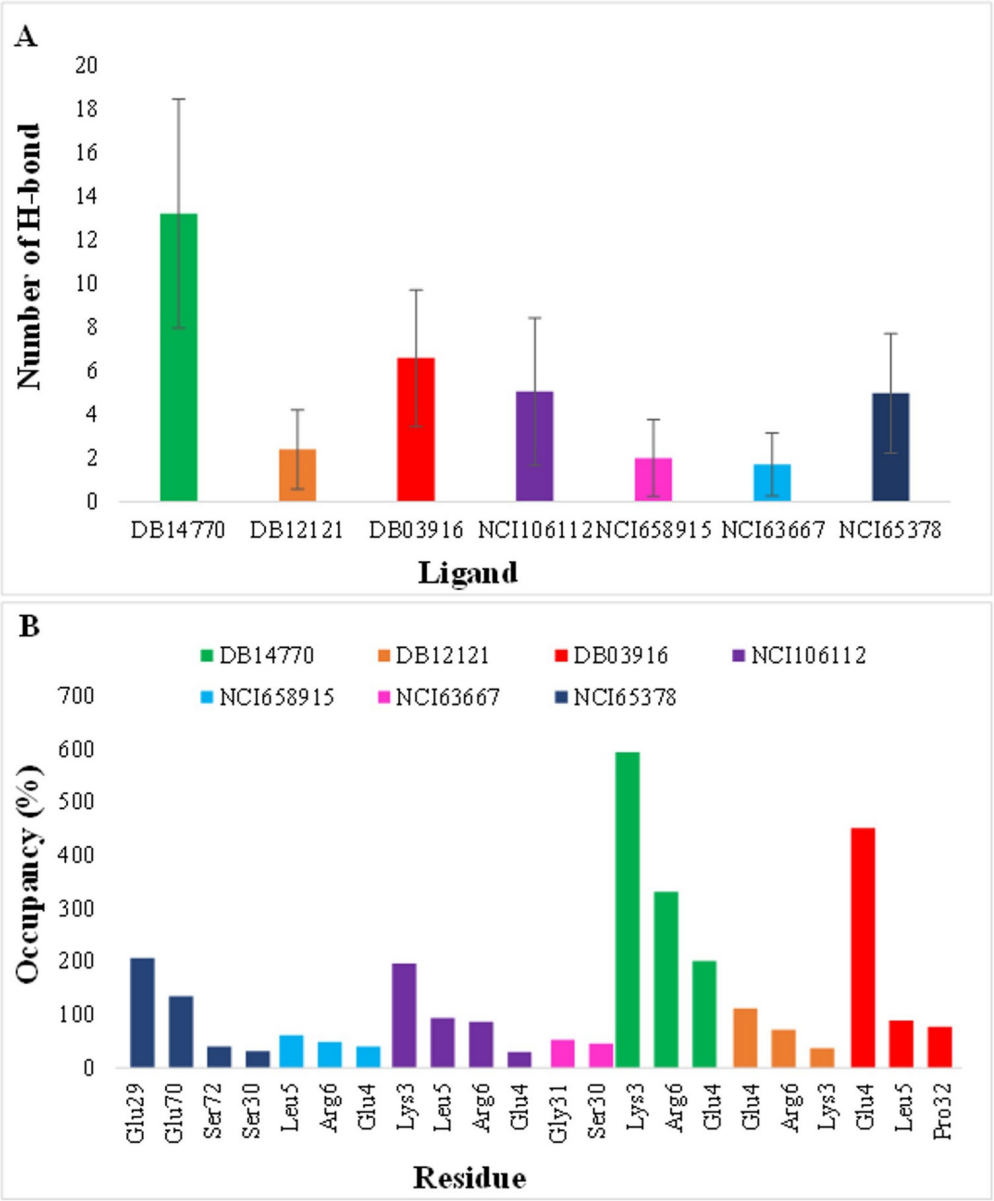
The hydrogen bond H-bond analysis. (A) H-bond average values of seven investigated compounds. (B) Hydrogen bond occupancy of 7 ligands by residues in 50 ns simulations trajectories, only interactions with a frequency greater than 30% were presented.

The number and occupancy of hydrogen bonds are crucial to determining the binding strength of the ligands at the active sites, with the H-bond occupation of > 75% defined as a strong hydrogen bond and ≥ 50% as a medium one [50]. Analysis of H-bond occupancy indicated that seven compounds established an average number of hydrogen bonds with residues in IL-8 (Fig 5A). DB14770 had the largest hydrogen bonds among these compounds. Moreover, IL-8’s Lys3 and Arg6 had the highest percentage of H-bond occupancy with DB14770 during the simulation time, and the key residue Glu4 also had a relatively high H-bond frequency of about 200% with this ligand (Fig 5B). Meanwhile, DB03916 established an average of 6.5 hydrogen bonds and high occupancy of two crucial residues, Glu4 and Leu5 of IL-8 (approximately 452% and 99%, respectively). Thus, DB03916 could have a good potency in binding to IL-8. NCI106112 and NCI65378 created an average of 5 hydrogen bonds with IL-8 protein. However, the major contributors to H-bond formation with NCI65378 were residues Glu29, Glu70, Ser72, and Ser30, and they were outside the active site of IL-8. At the same time, NCI106112 was found to interact with the two hot-spot amino acids (> 75%) highly. This result strongly supported the greater binding strength of the active sites of IL-8 to only NCI106112. Similarly, DB12121 also interacted with Glu4 and Arg6 by strong H-bonds. On the other hand, NC63667 and NCI658915 created a merely average of two hydrogen bonds and a low H-bond percentage with key residues (< 50%) throughout the simulation.

Overall, with three critical parameters to validate the results of molecular dynamics simulations, the analysis demonstrated that DB03916, DB12121, DB14770, and NCI106112 could form H-bond to crucial residues of IL-8 with high occupancies. Their RMSD and RMSF values were within the allowed range. This result signified the stability of the protein-ligand complexes.

#### 100 ns MD simulations to identify the best compounds

Complexes and apo forms of IL-8 with DB14770, DB12121, DB03916, and NCI106112 were simulated in a longer time (100 ns) to investigate the potential of stable binding at an active site. The apostates and the protein complexes with DB12121 and DB03916 had acceptable stability with their RMSD carbon backbone fluctuations of ≤ 3 Å. The IL-8 DB14770 complex was quite stable in the first half of the MD simulations and the last 30 ns. However, during the 50–70 ns period, the RMSD value increased significantly. When complexed with NCI106112, IL-8 protein had an RMSD rising for up to 30 ns and became relatively stable near the end of the simulation.

**Fig 6:**
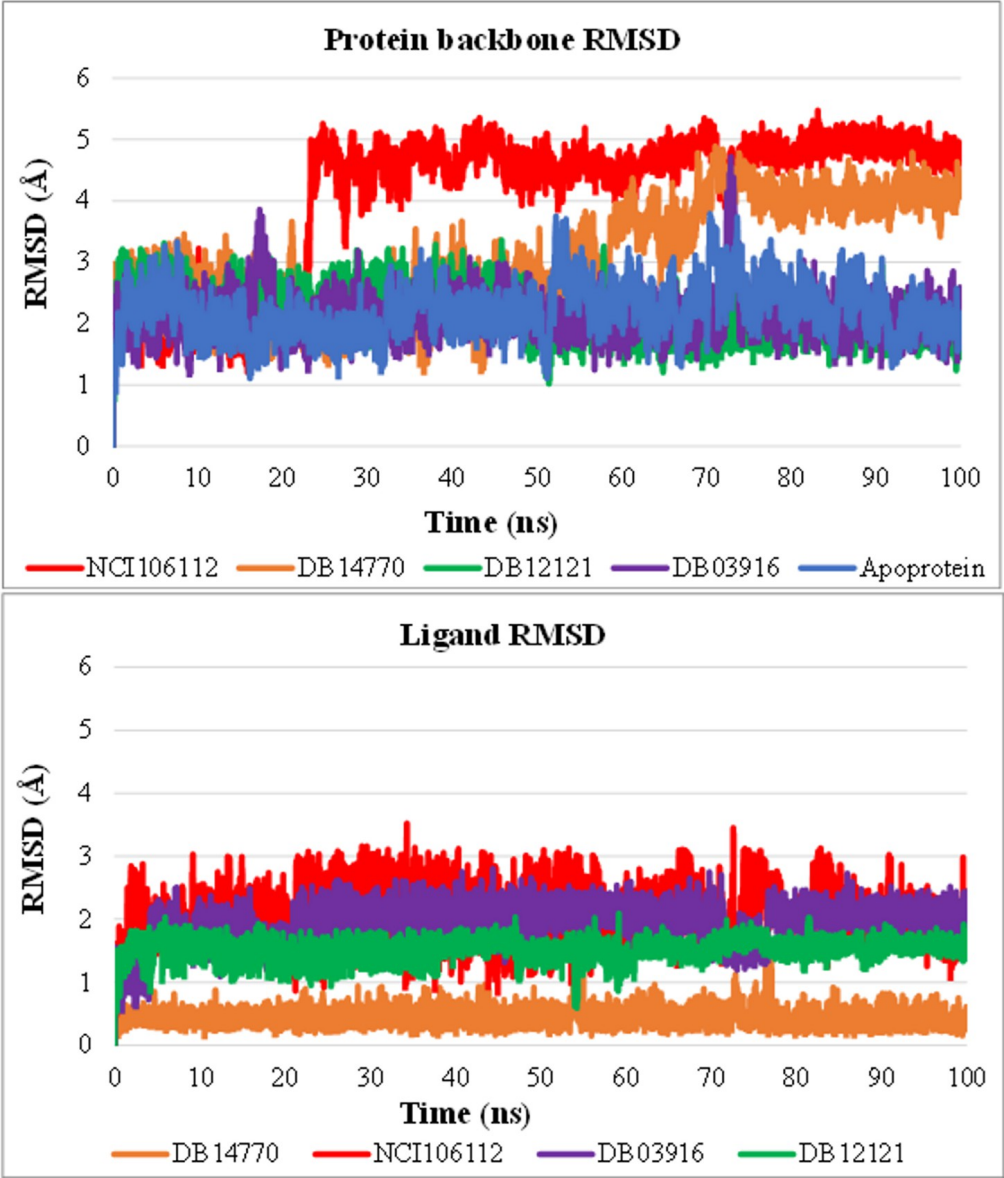
RMSD of (A) protein backbone and (B) ligands of complexes from 100 ns MD trajectories.

Additionally, to take a closer look at the effect of ligands on IL-8 protein, the RMSD of ligands was also examined. DB14770, DB12121, and DB03916 were in the range fluctuation of about 1 Å, which could be observed over the simulation time, as shown in Fig 6B. The NCI106112 compound is considered to be less stable than the three above ligands, with a deviation in a range of 2.5 Å.

**Fig 7:**
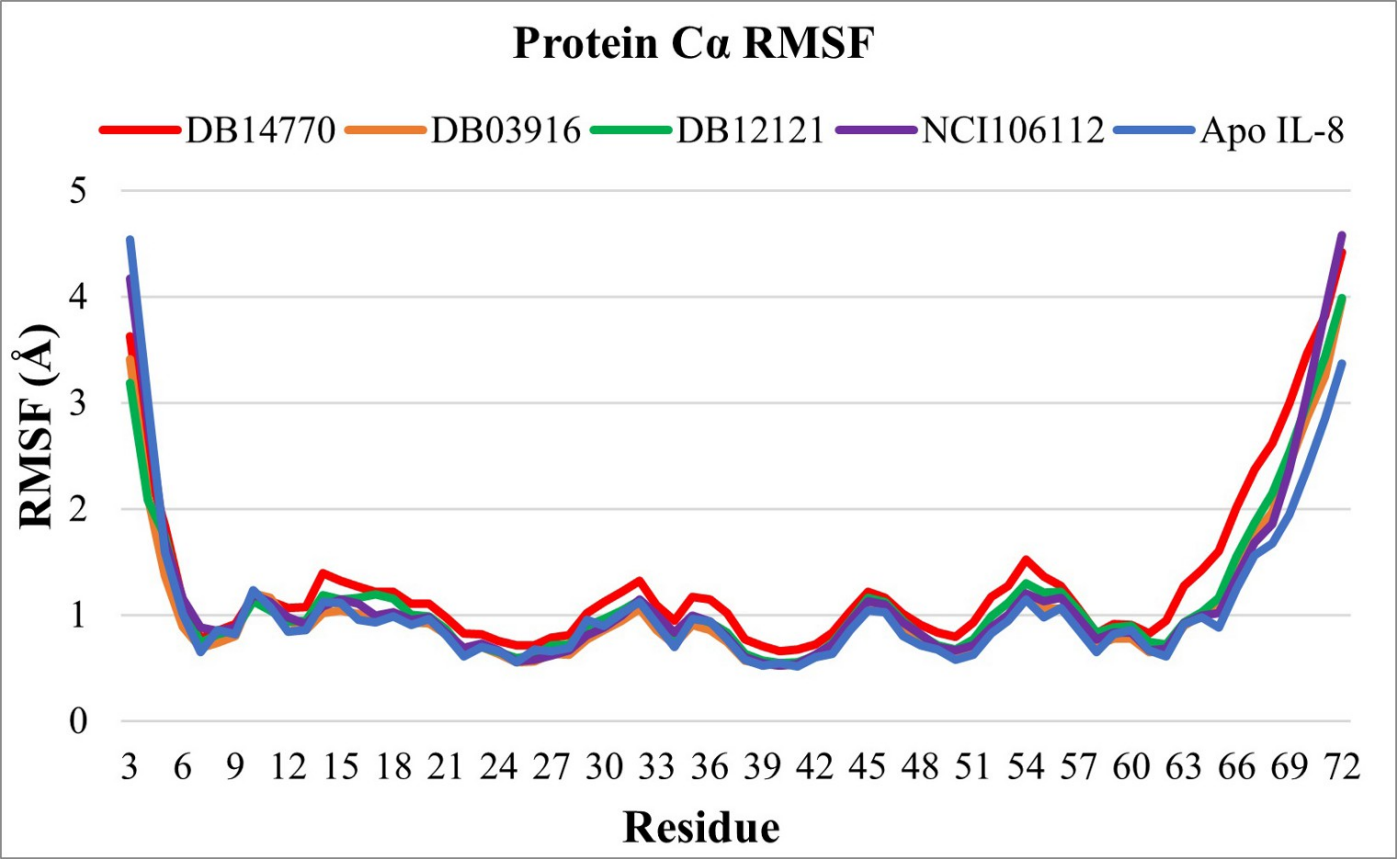
C-alpha RMSF analysis for IL-8 in apo and holo states.

The RMSF analysis showed that most residues have an RMSF value of less than 2 Å, except for Lys3 and residues 68−72 at the N and C termini regions (Fig 7). As previously mentioned, the higher mobility of residues in these regions was also described proven in another study [47]. These results indicated that the structures fluctuated around a stable average conformation.

**Fig 8:**
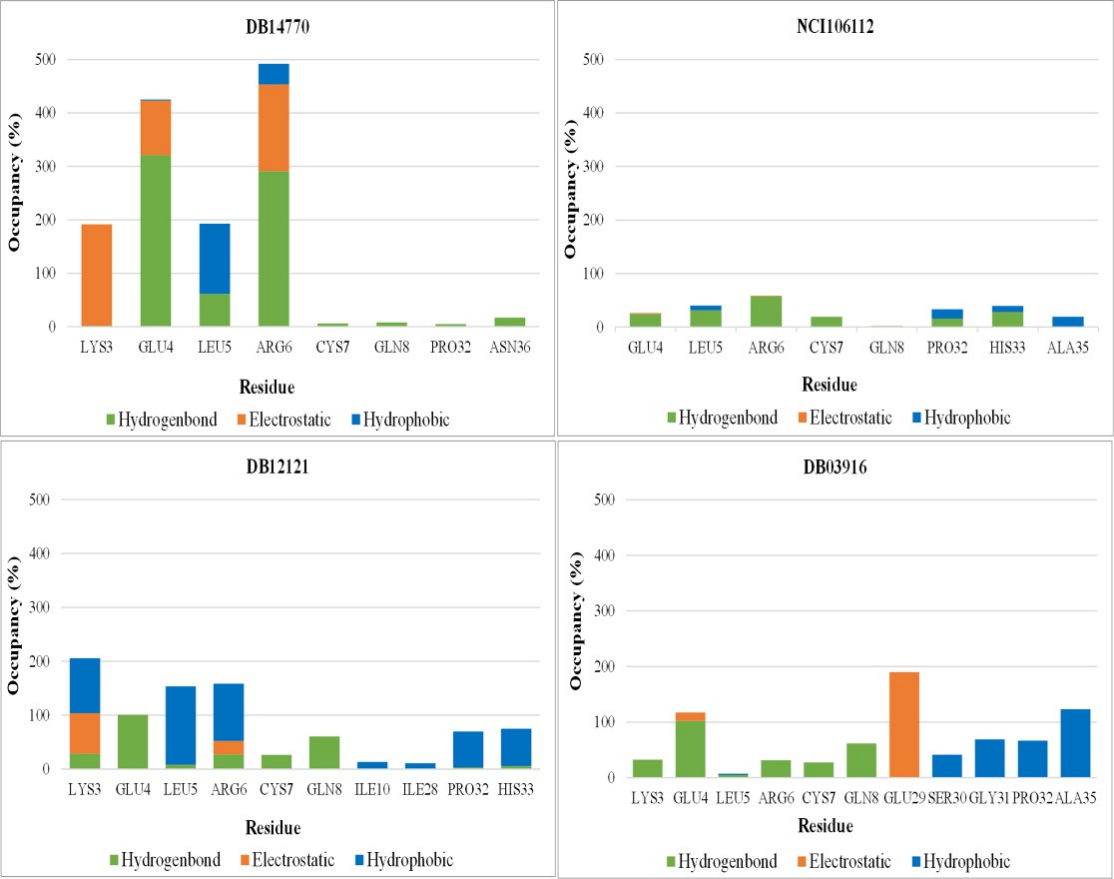
The interactions occupancy between ligands and the residues of IL-8.

The ligand DB14770 exhibited the highest frequency of total interactions, followed by DB12121, DB03916, and NCI106112 (Fig 8). Detailed calculations identified Glu4 and Arg6 as residues strongly H-bonded to DB14770 with a high frequency of 322% and 290%, respectively. These amino acids also had ionic interactions with the occupation of more than 100%. In addition, DB14770 was found to interact with the key residue Leu5 (131%) by hydrophobic contact and one non-hot-spot Lys3 (190%) through electrostatic interaction. The analysis indicated that DB14770 took part in the number of interactions, especially establishing potent hydrogen bonds with IL-8 active site residues, and the binding strength of this compound was outstanding.

The compound DB12121 formed hydrogen bonds, electrostatic and hydrophobic interactions with Arg6 of IL-8; the occupancies were approximately 27%, 25%, and 107%, respectively. Meanwhile, DB12121 strongly interacted with Glu4 by only H-bond (101%). The ability of DB12121 to form pi–alkyl bonds with Leu5 was the highest of four investigated ligands. Additionally, four in ten amino acids that interacted with DB12121 were non-hot-spot residues of IL-8: are Lys3, Gln8, Pro32, and His33. However, this compound’s interactions with these residues accounted for a significant portion of total interactions. The results of the interaction analysis suggested that DB12121 might be a promising inhibitor for IL-8.

The binding of DB03916 to IL-8’s Glu4 was found to be stabilized by a strong H-bond (102%). Nevertheless, the frequency of interactions between this ligand and two crucial residues, Leu5, Arg6 at the binding site, was too low. By contrast, DB03916 was bound to other amino acids of IL-8, namely Gln8, Glu29, Ser30, Gly31, Pro32, and Ala35, with more than 50% percentage. Thus, it was considered the binding potency at the active site of this ligand lower than that of DB14770, DB12121.

**Fig 9:**
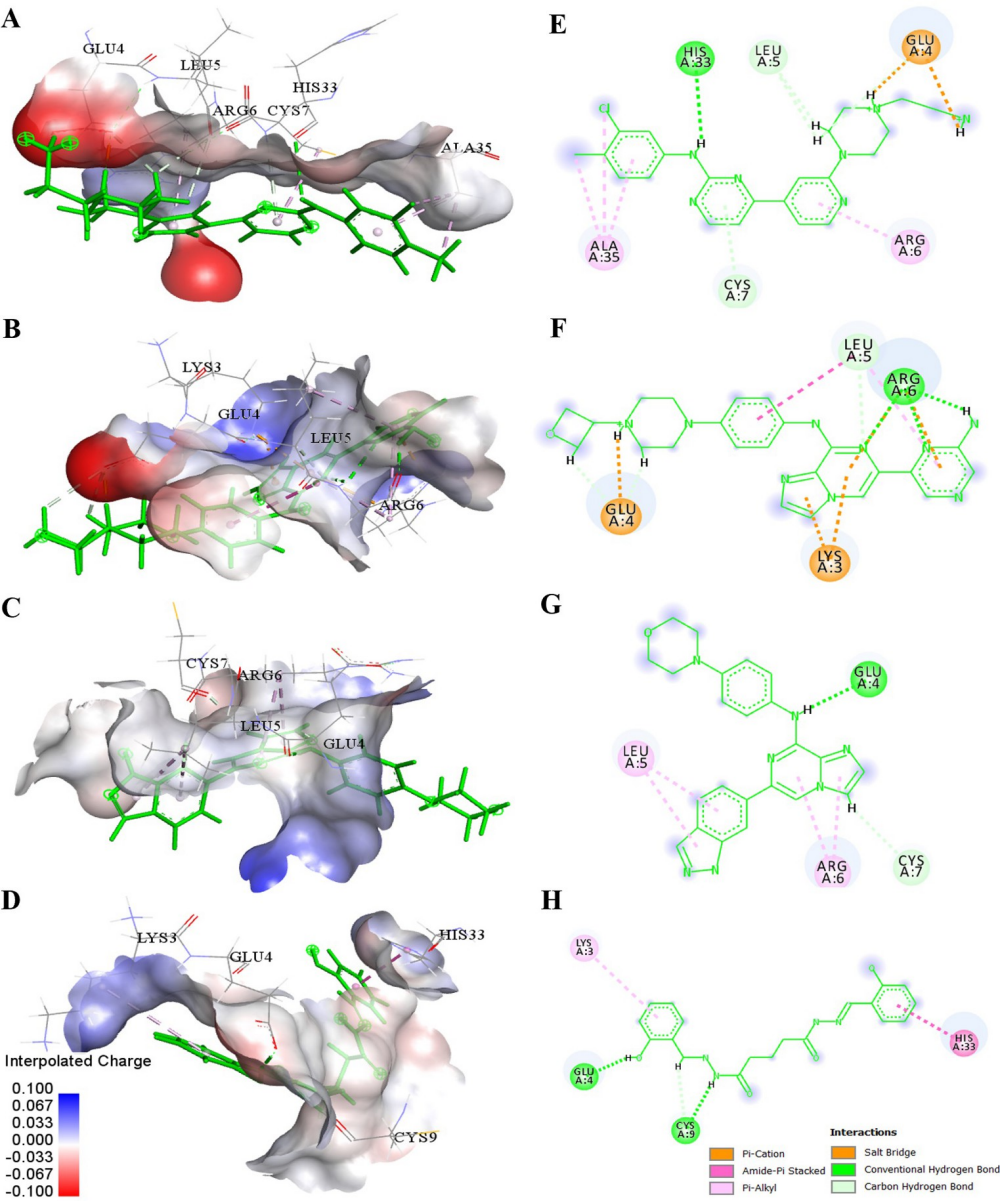
Schematic representations (3D and 2D) of the binding interactions between the IL-8 active site and (A & E) DB14770, (B & F) DB12121, (C & G) DB03916, (D & H) NCI106112 at 100 ns of the MD simulations.

In comparison to the three above compounds, NCI106112 created much fewer interactions with the ELR motif of IL-8. The highest occupancy observed was 58% of hydrogen bonding with IL-8’s Arg6, and the proportion of other interactions with mentioned residues in the plot was minimal. This demonstrated NCI106112 had the weakest binding capability on target in four compounds. The 3D and 2D diagrams in Fig 9 clearly illustrated the four best ligand atoms’ interaction with the IL-8 residues at the end of 100 ns of the MD simulations.

Generally, analysis of RMSD, RMSF values, H-bond formation, and other interactions with IL-8 protein revealed that DB14770, DB12121, and DB03916 compounds established high structural stability and binding potential with IL-8 active site key residues. Their free binding energies were further calculated.

#### Free binding energy

In the molecular dynamic simulation, free energy calculation takes a significant role in determining the binding energy of the ligand-protein complex. In this work, the g_mmpbsa tool of Gromacs was used to calculate binding energy. The ligand and the protein created a variety of interactions, including hydrophobic, hydrogen, electrostatic, and other interactions. Each of the individual interactions contributed either positively or negatively to the overall binding free energy. The binding energy of all compounds had negative scores (Table 3), which demonstrated a good affinity for IL-8. Among the three hits, DB14770 had the most negative binding free energy in the range of −488.998 kJ/mol. This is followed by DB12121, and DB03916, with the total binding energy values of −89.698 and −26.981 kJ/mol, respectively.

**Table 3:** Free energy calculations for the best ligands after MD simulations.

Energy terms (kJ/mol)	DB03916	DB12121	DB14770
**van der Waal energy** (***E***_***vdw***_)	−77.287 ± 0.252	−67.334 ± 0.278	−76.460 ± 0.192
**Electrostatic energy** (***E***_***ele***_)	−281.433 ± 3.899	−44.379 ± 0.443	−903.067 ± 1.047
**Polar solvation energy** (***G***_***polar***_)	344.398 ± 1.688	30.663 ± 0.472	503.504 ± 0.661
**SASA energy** (***G***_***nonpol***_)	−12.536 ± 0.022	−8.626 ± 0.034	−12.997 ± 0.012
**Binding energy** (Δ***G***_***bind***_)	−26.981 ± 2.286	−89.698 ± 0.387	−488.998 ± 0.440

The Δ*G*_*bind*_ can be divided into polar (*E*_*ele*_ + *G*_*polar*_) and nonpolar (*E*_*vdw*_ + *G*_*nonpol*_) energies. The free energy of IL-8 binding was primarily contributed from the *E*_*vdw*_ + *G*_*nonpol*_ for DB03916, while *E*_*ele*_ + *G*_*polar*_ showed a high unfavorable positive energy of contribution. Thus, the free binding energy of this compound was higher than the two remaining complexes. Generally, the binding free energies values were clearly low, especially for DB14770. One explanation might be the contribution of the electrostatic energy, which is highly influenced by the dielectric constant (ε). The dielectric constant’s value depends on the binding site’s characteristics between the protein and each ligand. It has been suggested that ε should be varied with the protein or the ligand residues instead of using the default parameter [44]. However, for the sake of simplicity, a default dielectric constant is usually used for the whole solute in the Poisson–Boltzmann models [42]. Hence, in our study, *E*_*ele*_ was calculated with a dielectric constant of unity (ε = 2). Additionally, the MM-PBSA approach was used to estimate the binding energy of top hits to give a comparable prediction of these compounds.

**Fig 10:**
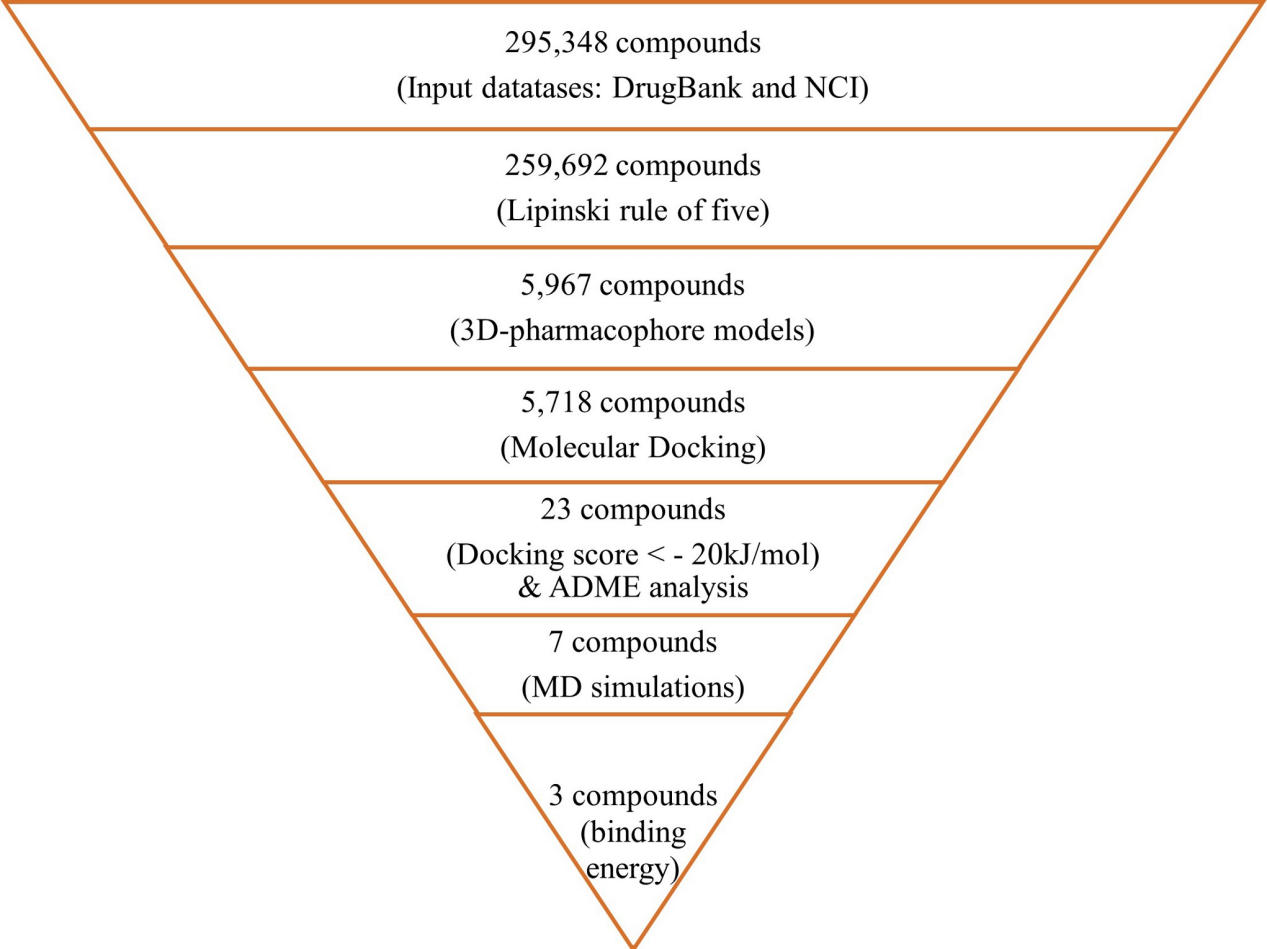
Summarize the studied results.

In summary, by analyzing the MD simulation process and calculating free binding energy, the best compounds were identified as DB14770, DB03916, and DB12121. They may block the protein-protein interaction between IL-8 and its receptor CXCR2, hence could be potential inhibitors for IL-8. The results of virtual screening, ADME properties prediction, and MD simulations are summarized in Fig 10.

## Conclusion

The role of IL-8 and its CXCR2 receptor in inflammations and human melanoma’s metastasis has become clear in recent years, rendering IL-8/CXCR2 an attractive drug target. Through computer-assisted drug design, we aimed to search for compounds that can bind to IL-8 at the IL-8/CXCR2 interface, thereby inhibiting the interaction between the two proteins. This may be an effective strategy to modulate the IL8-involved signaling pathway, providing potential therapy in related diseases. This *in silico* screening will enhance the hit rate and reduce the *in vitro* and *in vivo* experimental time and cost. The virtual screening by structure-based pharmacophore and molecular docking models was carried out with large libraries to identify potential drug candidates capable of binding to IL-8. We have identified 23 compounds having docking scores <–20kJ/mol that interacted with key residues of IL-8. Subsequent application of the SwissADME tool resulted in seven druggable candidates with suitable pharmacokinetic properties. MD simulation during 100 ns was further executed, revealing that DB14770, DB12121, and DB03916 could form stable complexes with IL-8. The negative binding energy finally confirmed their structural stability and effective binding affinities for IL-8. These potential compounds can be developed into novel IL-8 inhibitors.

## Supporting information

S1 Fig The detail interactions of docking results. Interactive models of top 23 ligands.
(TIF)

S2 Fig The RMSD results throughout the course of 50 ns MD simulations. RMSD of IL-8 in apoprotein and in complexes with the seven ligands (in blue) and RMSD profiles of the corresponding ligands (in orange).
(TIF)

S3 Fig RMSF output along the 50 ns MD trajectory. Carbon alpha RMSF values of IL-8 in the apoprotein and its complexes with seven ligands.
(TIF)

S1 Table Computed physicochemical properties of top ligands. Including descriptors: molecular weight, rotatable bonds, H-bond, LogP and LogS.
(PDF)

S2 Table Predicted the metabolism by some major CYP450 enzymes of top ligands. Five major isoforms (CYP1A2, CYP2C19, CYP2C9, CYP2D6, CYP3A4).
(PDF)
