# Supplementary figures and images for: Identification of potential interleukin-8 inhibitors acting on the interactive site between chemokine and CXCR2 receptor: A computational approach
Source: PLoS One. 2022 Feb 24;17(2):e0264385. doi: 10.1371/journal.pone.0264385 (PMC8870564; doi:10.1371/journal.pone.0264385)

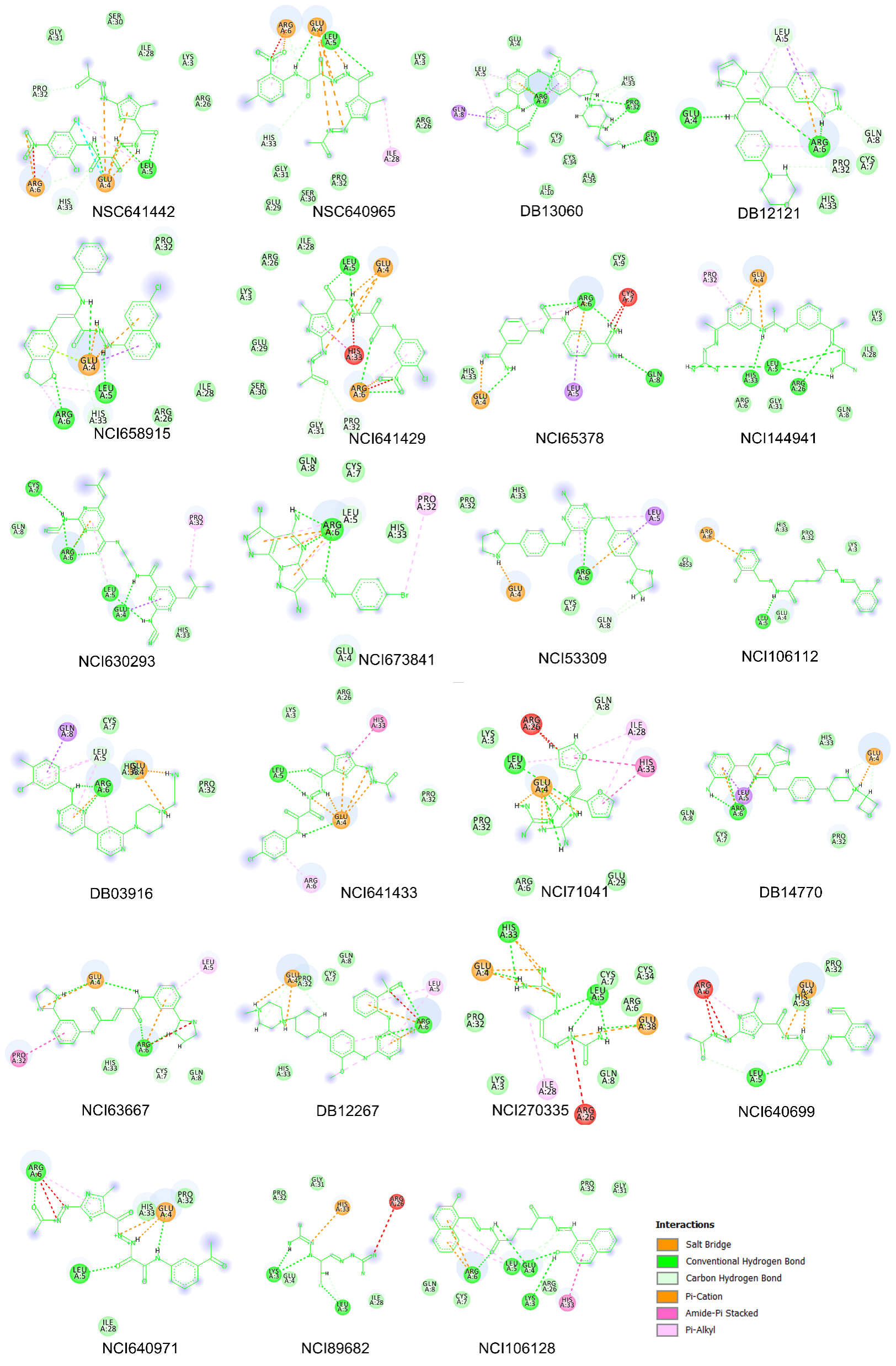

Supplement: S1 Fig — Interactive models of top 23 ligands. (TIF) [file pone.0264385.s001.tif]

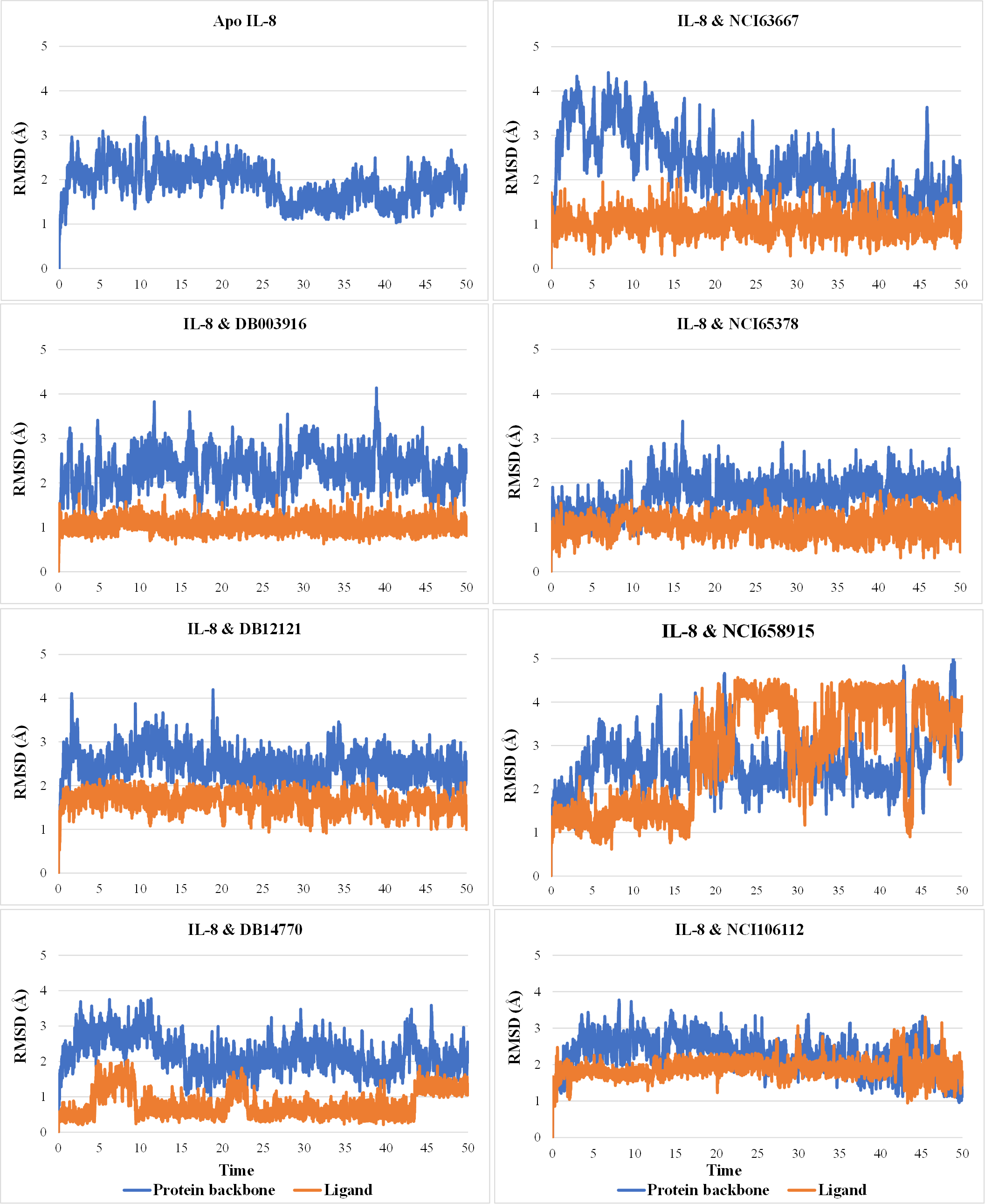

Supplement: S2 Fig — RMSD of IL-8 in apoprotein and in complexes with the seven ligands (in blue) and RMSD profiles of the corresponding ligands (in orange). (TIF) [file pone.0264385.s002.tif]

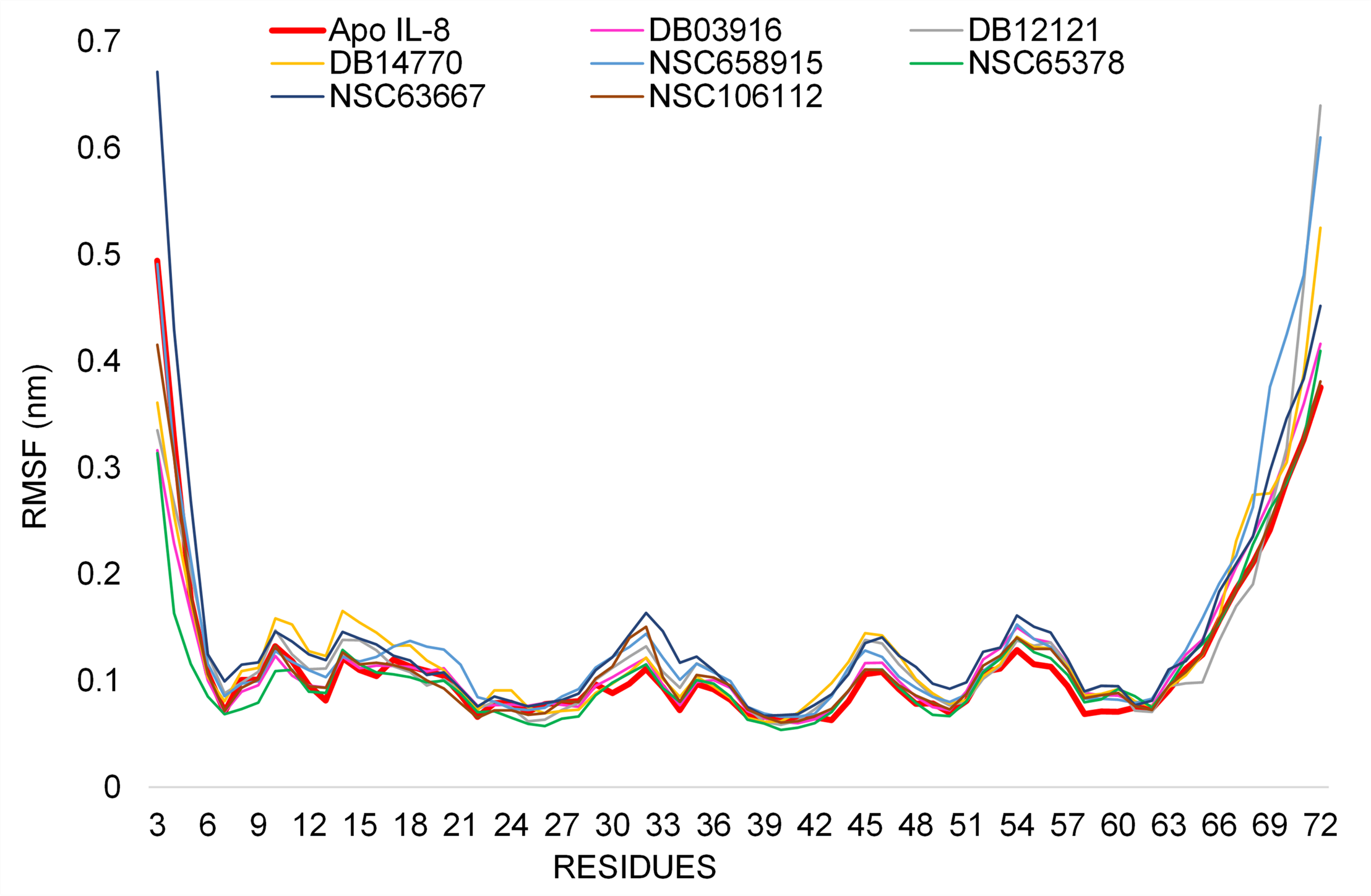

Supplement: S3 Fig — Carbon alpha RMSF values of IL-8 in the apoprotein and its complexes with seven ligands. (TIF) [file pone.0264385.s003.tif]
