# Supplementary material for: Identification of potential interleukin-8 inhibitors acting on the interactive site between chemokine and CXCR2 receptor: A computational approach
Source: PLoS One. 2022 Feb 24;17(2):e0264385. doi: 10.1371/journal.pone.0264385 (PMC8870564; doi:10.1371/journal.pone.0264385)
Supplement: S1 Table — Including descriptors: molecular weight, rotatable bonds, H-bond, LogP and LogS. (PDF) [file pone.0264385.s004.pdf]

S1 Table. Computed physicochemical properties of top ligands.

Rank	Ligand ID	Physicochemical properties					Lipid solubility	Water solubility	
		Molecular weight[a]	Rotatable bonds	H-bond acceptors	H-bond donors	TPSA[b]	Consensus LogP	ESOL LogS	ESOL Class
1	NCI640971	418.43	11	6	5	186.63	0.86	−3.12	Soluble
2	NCI640965	435.41	11	7	5	215.38	0.38	−3.89	Soluble
3	DB13060	580.12	10	7	4	114.88	3.64	−6.01	Poorly soluble
4	DB12121	411.46	4	4	2	83.37	2.41	−4.97	Moderately soluble
5	NCI144941	424.53	8	4	6	209.67	1.49	−2.64	Soluble
6	NCI65378	294.31	5	4	3	93.50	0.53	−1.66	Very soluble
7	NCI641429	455.83	11	7	5	215.38	0.73	−3.84	Soluble
8	NCI53309	414.47	6	5	5	137.53	1.39	−3.26	Soluble
9	NCI630293	460.49	11	8	2	181.40	1.83	−4.42	Moderately soluble
10	NCI641442	490.28	11	7	5	215.38	1.01	−4.43	Moderately soluble
11	NCI673841	387.20	2	5	4	161.65	1.62	−4.09	Moderately soluble
12	DB14770	443.50	5	6	2	109.73	1.08	−3.61	Soluble
13	NCI658915	486.91	8	5	3	101.58	4.04	−6.45	Poorly soluble
14	DB03916	423.94	6	5	2	83.20	2.75	−4.40	Moderately soluble
15	NCI63667	402.45	8	4	4	106.98	1.26	−2.17	Soluble
16	NCI641433	410.84	10	5	5	169.56	1.47	−3.76	Soluble
17	NCI89682	199.19	6	5	6	144.90	2.00	0.86	Highly soluble
18	NCI71041	300.28	7	6	4	127.10	2.00	0.86	Highly soluble
19	NCI106128	468.50	10	6	4	123.38	2.00	0.86	Highly soluble
20	NCI270335	184.18	5	4	4	117.89	1.42	0.53	Highly soluble
21	DB12267	584.09	8	6	2	95.67	3.79	−6.20	Poorly soluble
22	NCI106112	368.39	10	6	4	123.38	1.96	−2.82	Soluble
23	NCI640966	401.40	10	6	5	193.35	0.83	−3.47	Soluble

[a]*Molecular weight: g/mol.*

[b]*TPSA (topological polar surface area): Å.*
