# Supplementary material for: Identification of potential interleukin-8 inhibitors acting on the interactive site between chemokine and CXCR2 receptor: A computational approach
Source: PLoS One. 2022 Feb 24;17(2):e0264385. doi: 10.1371/journal.pone.0264385 (PMC8870564; doi:10.1371/journal.pone.0264385)
Supplement: S2 Table — Five major isoforms (CYP1A2, CYP2C19, CYP2C9, CYP2D6, CYP3A4). (PDF) [file pone.0264385.s005.pdf]

S2 Table. Predicted the metabolism by some cytochrome (CYP) enzymes of top ligands.

Rank	Ligand ID	CYP1A2	CYP2C19	CYP2C9	CYP2D6	CYP 3A4
1	NCI640971	No	No	No	No	Yes
2	NCI640965	No	Yes	No	No	Yes
3	DB13060	No	Yes	No	Yes	Yes
4	DB12121	Yes	Yes	Yes	Yes	Yes
5	NCI144941	No	No	No	No	No
6	NCI65378	No	No	No	No	No
7	NCI641429	No	Yes	No	No	Yes
8	NCI53309	Yes	No	No	No	Yes
9	NCI630293	No	Yes	Yes	No	Yes
10	NCI641442	No	Yes	No	No	Yes
11	NCI673841	Yes	Yes	No	No	No
12	DB14770	No	No	Yes	Yes	Yes
13	NCI658915	No	Yes	Yes	No	Yes
14	DB03916	Yes	No	No	Yes	Yes
15	NCI63667	No	No	No	No	No
16	NCI641433	No	No	No	No	Yes
17	NCI89682	No	No	No	No	No
18	NCI71041	No	No	No	No	No
19	NCI106128	No	No	No	No	No
20	NCI270335	No	No	No	No	No
21	DB12267	No	Yes	No	Yes	No
22	NCI106112	No	No	No	No	No
23	NCI640966	No	No	No	No	Yes
